# Potential therapeutic targeting of BK_Ca_ channels in glioblastoma treatment

**DOI:** 10.1002/1878-0261.70167

**Published:** 2025-12-05

**Authors:** Kamila Maliszewska‐Olejniczak, Karolina Pytlak, Sandra Jaworowska, Bogusz Kulawiak, Piotr Bednarczyk

**Affiliations:** ^1^ Department of Physics and Biophysics Institute of Biology, Warsaw University of Life Sciences – SGGW Warsaw Poland; ^2^ Laboratory of Intracellular Ion Channels, Nencki Institute of Experimental Biology, Polish Academy of Sciences Warsaw Poland

**Keywords:** BK_Ca_ channel, gBK_Ca_, glioblastoma multiforme, mitoBK_Ca_, oncochannels, therapeutic targeting

## Abstract

Potassium channels in brain tissue orchestrate essential cellular processes, including the regulation of membrane potential and neuronal excitability. Among them, large‐conductance calcium‐activated potassium (BK_Ca_) channels play a pivotal role in both normal brain physiology and the pathogenesis of glioblastoma multiforme, a highly aggressive primary brain tumor. Within the central nervous system, BK_Ca_ channels are widely expressed in neurons, astrocytes, and oligodendrocytes, contributing to ion homeostasis and synaptic transmission. In glioblastoma cells, overexpression of BK_Ca_ channels, particularly the glioma‐specific gBK_Ca_ variant, facilitates tumor progression by enhancing cell migration, invasion, and therapeutic resistance. Recent evidence highlights the significance of the mitochondrial isoform of the BK_Ca_ channel (mitoBK_Ca_) in modulating oxidative phosphorylation and reactive oxygen species generation, thereby promoting tumor cell survival under hypoxic and cytotoxic stress. This review summarizes current insights into the role of BK_Ca_ and mitoBK_Ca_ channels in glioblastoma biology, their potential classification as oncochannels, and the emerging pharmacological strategies targeting these channels, emphasizing the translational challenges in developing BK_Ca_‐directed therapies for glioblastoma treatment.

## Graphical abstract

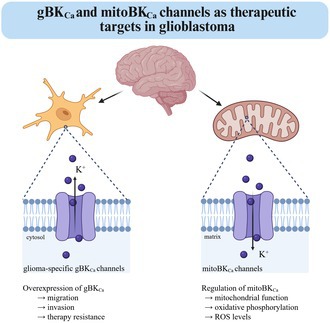

**Abbreviations**
AQP4: aquaporin‐4
BBB: blood–brain barrier
BK_Ca_
: large‐conductance Ca^2+^‐activated K^+^ channel (KCa1.1)
BTIC: brain tumor–initiating cell
CaMKII: calcium/calmodulin‐dependent protein kinase II
Cav (VGCC): voltage‐gated Ca^2+^ channels
ClC3: chloride channel 3
CRAC: calcium release‐activated calcium channel
CXCL12 (SDF‐1): C‐X‐C motif chemokine ligand 12
ECM: extracellular matrix
EMT: epithelial–mesenchymal transition
ER: endoplasmic reticulum
ERK1/2: extracellular signal‐regulated kinases 1/2
gBK_Ca_
: glioma‐specific BK_Ca_ splice variant
GBM: glioblastoma multiforme
GSCs: glioblastoma stem cells
IbTX: iberiotoxin (selective BK_Ca_ blocker)
IDH1/IDH2: isocitrate dehydrogenase 1/2
IK_Ca_ (KCa3.1): intermediate‐conductance Ca^2+^‐activated K^+^ channel
IP3R: inositol‐1,4,5‐trisphosphate receptor
K_ATP_
: ATP‐sensitive K^+^ channel
K_Ca_
: Ca^2+^‐activated K^+^ channel family
KCNMA1: gene encoding the BK_Ca_ α‐subunit
Kir_4.1_
: inward‐rectifier K^+^ channel 4.1
LOF: loss‐of‐function
mitoBK_Ca_
: mitochondrial BK_Ca_ channel
MPTPP: mitochondria‐targeting triphenylphosphine‐modified polymer
NCX: Na^+^/Ca^2+^ exchanger
NS004: synthetic BK_Ca_ activator
NS11021: synthetic BK_Ca_ activator
NS1619: synthetic BK_Ca_ activator
OP‐A: ophiobolin A
Orai1: pore‐forming CRAC subunit mediating SOCE
p53: tumor suppressor protein p53
Pax: paxilline (BK_Ca_ blocker)
PGD: pore–gate domain (S5–S6)
PI3K: phosphoinositide 3‐kinase
PKA: protein kinase A
PKC: protein kinase C
RAS: rat sarcoma oncogene pathway
RB: retinoblastoma protein
RCK1/RCK2: regulator of conductance for K^+^ domains 1/2
ROS: reactive oxygen species
RTK: receptor tyrosine kinase
RyR: ryanodine receptor
SK: small‐conductance Ca^2+^‐activated K^+^ channel
SOC: store‐operated Ca^2+^ channel
TEA: tetraethylammonium
TMZ: temozolomide
TRPC1: transient receptor potential canonical 1
TRPM8: transient receptor potential melastatin 8
TRPV1: transient receptor potential vanilloid 1
VEDEC: BK_Ca_ splice variant targeted to mitochondria
VGCCs: voltage‐gated calcium channels
VSD: voltage‐sensing domain

## 1 Introduction

Glioblastoma, also known as glioblastoma multiforme (GBM), is the most aggressive and common form of primary brain tumor in adults, classified as a grade IV glioma by the World Health Organization (WHO) [1, 2]. Histopathological features include necrosis and endothelial proliferation. GBM can arise from lower‐grade gliomas (grades II or III) and is characterized by rapid growth, invasiveness, and a poor prognosis [1, 2]. Common molecular changes include mutations in genes regulating receptor tyrosine kinase (RTK)/rat sarcoma (RAS)/phosphoinositide 3‐kinase (PI3K), p53, and retinoblastoma protein (RB) signaling pathways [2]. *IDH1* or *IDH2* mutations are associated with secondary glioblastomas, which generally have a more favorable outcome [2]. Standard treatment of GBM involves surgical resection, radiotherapy, and chemotherapy with temozolomide [1, 2]. Despite aggressive treatment, the median survival time is ~12–15 months, with a 5‐year survival rate of only 6.7% [3]. Challenges in treatment include drug resistance, tumor recurrence, and the blood–brain (BBB) barrier limiting drug delivery [3]. GBM is a highly heterogeneous tumor, with genetic diversity, glioblastoma stem cells (GSCs), and a complex tumor microenvironment, all contributing to therapeutic resistance and recurrence [4]. Genetic variability leads to differential treatment responses and complicates standard therapies [5], while GSCs promote tumor progression and evade therapy through enhanced DNA repair and metabolic plasticity [4, 6, 7]. Additionally, the immunosuppressive microenvironment supports tumor survival and limits treatment efficacy [8].

Emerging research indicates that intracellular Ca^2+^ is a central regulator of glioma cell movement through multiple pathways, including activation of Ca^2^‐activated K^+^ (K_Ca_) channels. For instance, functional expression of BK_Ca_ channels in human glioma cells has been linked to migration [9, 10]. Moreover, CXCL12‐induced glioblastoma chemotaxis requires intermediate‐conductance K_Ca3.1_ channel activity [11], while store‐operated Ca^2+^ entry mediated by Orai1 has been shown to regulate glioma cell migration and invasion by modulating focal adhesion turnover and EMT‐like processes [12]. To reduce energy consumption and ensure precise regulation of intracellular Ca^2+^ levels, K_Ca_ channels and Ca^2+^ channels often form functional complexes in both excitable and nonexcitable cells [13, 14, 15, 16]. Interestingly, emerging evidence indicates that such potassium–calcium (both voltage‐gated and nonvoltage‐gated) channel complexes are also present in cancer cells, where they contribute to key cancer‐associated processes, including enhanced cell proliferation, increased migratory capacity, and the promotion of metastasis formation.

Among the ion channels implicated in glioblastoma pathophysiology, large‐conductance calcium‐ and voltage‐activated potassium (BK_Ca_) channels encoded by the *KCNMA1* gene play a significant role, because their expression levels correlate with the malignancy grade of the tumor. BK_Ca_ channels are activated by both membrane depolarization and intracellular Ca^2+^ elevation [17, 18, 19, 20]. This dual regulation allows BK_Ca_ channels to integrate electrical and calcium signaling, which is central to their physiological roles in various tissues. The gating mechanism of these channels is highly specialized and involves allosteric coupling between the voltage sensor and the Ca^2+^ binding sites. Specifically, the voltage sensor, primarily located in the S4 transmembrane domain, responds dynamically to changes in membrane potential, while the Ca^2+^ binding sites are situated in the large cytosolic C‐terminal domain, particularly within the RCK1 and RCK2 domains [21]. The binding of Ca^2+^ to these intracellular domains induces conformational changes that facilitate channel opening, thereby enhancing potassium conductance [22]. The structural organization of BK_Ca_ channels reflects their complex functional regulation. These channels are tetrameric structures composed of four α subunits, each containing seven transmembrane segments (S0–S6) [23]. Notably, the S0 segment is unique to BK_Ca_ channels and gives rise to an external NH₂‐terminus [24]. Voltage‐sensing domain (VSD, segments S0–S4) and a pore–gate domain (PGD, segments S5–S6), are both embedded in the plasma membrane [25]. In the membrane topology, the transmembrane helices are arranged in an alternating orientation, with every second loop segment facing either the extracellular or intracellular side. Together, the α subunits form the pore through which K^+^ passes. Additionally, the cytosolic domain, which includes the RCK1 and RCK2 domains, forms a gating ring that is crucial for Ca^2+^ sensing and channel gating [21, 22]. Upon Ca^2+^ binding, this gating ring undergoes structural rearrangements that are essential for channel activation [21, 22], providing a direct structural basis for the allosteric coupling of calcium sensing and pore opening. However, in the case of the mitochondrial isoform (mitoBK_Ca_), the same topology is preserved relative to the inner mitochondrial membrane, meaning that the regions corresponding to ‘extracellular’ domains are actually facing the intermembrane space, while the C‐terminal Ca^2+^‐sensing RCK1/RCK2 domains are directed toward the mitochondrial matrix. This inverted spatial context has profound functional implications, as mitoBK_Ca_ channels respond to mitochondrial matrix rather than cytosolic Ca^2+^ signals [26]. A landmark study by the MacKinnon laboratory in 2017 resolved the cryo‐electron microscopy structure of the human BK_Ca_ channel, providing critical insights into its gating mechanism and structural organization [27]. This structural information has opened new avenues for the rational design of BK_Ca_ channel modulators, including agents that could interact with extracellular sites accessible from outside the cell membrane.

However, despite these advances in our understanding of BK_Ca_ channel architecture and function, the pathological roles of BK_Ca_ channels in cancer, particularly in glioblastoma, remain incompletely understood. Therefore, in this review we aim to bridge the gap between structural knowledge and pathophysiological relevance by discussing several key aspects of BK_Ca_ channel biology in the context of glioblastoma. Specifically, we will address the role of BK_Ca_ channels in brain tissue and their implications for glioblastoma multiforme pathology, examine the emerging concept of BK_Ca_ channels as oncochannels in glioblastoma, and highlight the contribution of mitoBK_Ca_ channels in glioma cells. Furthermore, we will explore pharmacological strategies aimed at modulating BK_Ca_ and mitoBK_Ca_ channels, summarize current therapeutic approaches targeting these channels in glioblastoma, and discuss the major challenges that must be overcome to develop effective BK_Ca_ channel‐targeted therapies for this aggressive brain tumor.

## 2 The role of BK_Ca_ channels in brain tissue

Calcium signaling is a critical regulator of various cellular processes that are essential for tumorigenesis, including proliferation, migration, apoptosis evasion, and therapy resistance. This signaling system involves a complex network of receptors, channels, pumps, exchangers, buffers, and sensors, many of which are altered in cancer cells [28]. Understanding the functions of BK_Ca_ channels in the healthy brain provides essential context for their role in tumor pathophysiology. BK_Ca_ channels are broadly distributed across multiple cell types in the brain, including neurons, astrocytes, and oligodendrocytes [13, 15, 29]. They are expressed in the cortex [30], hippocampus [31], cerebellum [32], and basal ganglia [33], among other brain parts [29]. By coupling cytosolic Ca^2+^ dynamics to membrane potential, BK_Ca_ channels regulate neuronal excitability and synaptic transmission, contribute to astrocytic potassium buffering, and support oligodendrocyte‐mediated ion homeostasis and myelination. This versatile regulation highlights the importance of BK_Ca_ channels in maintaining normal brain function, while in glioblastoma, their dysregulated activity and overexpression may enhance Ca^2+^ signaling dynamics and membrane potential control, thereby contributing to tumor aggressiveness [34]. Depending on the brain region and cell type, BK_Ca_ channels colocalize with voltage‐dependent calcium channels, NMDA receptors, and TRPV1 channels. Additionally, BK_Ca_ channels interact with other proteins responsible for calcium movement, such as ryanodine receptors (RyR) in the ER [35]. BK_Ca_ channels are intrinsically voltage‐gated, and their activation is facilitated by intracellular Ca^2+^, which shifts their voltage‐dependence toward more negative potentials, thereby enhancing their open probability at physiological membrane potentials. BK_Ca_ channels colocalize with store‐operated calcium channels (SOCs), such as Orai1, forming a feedback loop where calcium influx activates BK_Ca_ channels, leading to hyperpolarization and further calcium entry [36]. Similarly, BK_Ca_ channels are closely associated with voltage‐gated calcium channels, especially Ca_V1.3_, enabling rapid activation in response to depolarization [15]. The localization of BK_Ca_ channels is stabilized by cytoskeletal proteins, including CTN‐1/α‐catulin and DYB‐1/dystrobrevin, which anchor them near calcium entry sites. Disruption of Ca_V2_ channels alters BK_Ca_ positioning, highlighting the structural role of calcium channels in nanodomain organization [37, 38]. In Purkinje cells, BK_Ca_ clusters near IP3Rs integrate intracellular calcium release with membrane repolarization. This precise arrangement, regulated by calcium channel proximity and cytoskeletal scaffolding, is critical for fine‐tuning cellular excitability and calcium signaling.

The BK_Ca_ channel comprises not only the α subunit but also the auxiliary β1–4 and γ subunits, which are encoded by distinct genes [39]. The β4 subunit, predominant in the brain, pairs with the α subunit in regions such as the neocortex, hypothalamus, and cerebellum, often exerting effects on channel gating and decreasing excitability in specific neuronal populations [40, 41, 42]. In contrast, β2 subunits are present at lower levels but influence excitability, notably in pyramidal neurons [43]. In addition to β subunits, γ subunits, specifically γ3, have been identified in brain tissue [44].

Dysfunction of BK_Ca_ channels in the brain is associated with numerous neurological disorders. For example, abnormal BK_Ca_ channel activity can result in excessive neuronal excitability, as observed in various forms of epilepsy. Additionally, impaired BK_Ca_ channel function impacts motor coordination, leading to ataxia, especially in cases where mutations affect BK_Ca_ function in the cerebellum. BK_Ca_ channels also play a neuroprotective role by countering excessive Ca^2+^ influx, and their dysfunction may contribute to diseases like Parkinson's and Alzheimer's, where oxidative stress and calcium dysregulation are prevalent. Loss‐of‐function (LOF) in BK_Ca_ channels contributes to neuropathic pain [45]. The functional consequences of BK_Ca_ channel dysfunction are complex and may vary, depending on the cellular context. These channels can both increase and decrease neuronal excitability, making their precise role in epilepsy controversial and a subject of ongoing investigation [46]. As such, understanding the bidirectional effects of BK_Ca_ channels is critical for the development of targeted therapies.

## 3 BK_Ca_ channel as oncochannel in glioblastoma

### 3.1 Ion channels and oncogenesis: From excitability to cancer progression

Ion channels are fundamental to the nervous system's electrical signaling, enabling the generation of neuronal action potentials through controlled ionic currents. This fundamental concept was first demonstrated by Hodgkin and Huxley in 1952, who quantitatively described how voltage‐gated Na^+^ and K^+^ channels produce the action potential in the squid giant axon [47]. Beyond their physiological roles, accumulating evidence indicates that the activity and expression of ion channels contribute to oncogenesis and tumor progression, including in glioblastoma. Several studies have indicated abnormal K^+^ channel expression in malignant tumors, as reviewed recently [48]. Beyond their roles in excitability, ion channels—including BK_Ca_—are increasingly linked to DNA damage signaling and cellular stress responses [49, 50, 51]. Overexpression of a particular splice variant of the BK_Ca_ is associated with the glioma malignancy level [52]. Moreover, the BK_Ca_ channel is overexpressed in other cancer types: bone, breast, ovary, and prostate; therefore, this channel is named an “oncochannel” [53]. Oncochannels play a role in glioblastoma stem cell characteristics, orchestrate and facilitate cell migration and brain infiltration, and control the cell cycle, leading to therapy resistance [37, 38].

### 3.2 Glioma‐specific BK_Ca_ splice variants and tumor selectivity

BK_Ca_ channels are widely expressed in excitable tissues, including the brain, heart, and muscles [54]. Therefore, nonselective modulation of these channels may cause systemic side effects such as cardiovascular, respiratory, or neuromuscular complications. In human glioma cell lines and glioblastoma multiforme tissues, however, a distinct splice variant of the BK_Ca_ channel—known as the glioma BK (gBK_Ca_) channel—is predominantly expressed. This isoform includes a unique 34 amino acid insertion in the intracellular region of the BK_Ca_ channel [55]. This glioma‐specific splice variant has been shown to contribute to glioma cell growth and migration, which likely underlies the highly aggressive behavior of these tumors [52, 55]. gBK_Ca_ channels are activated by calcium signals from IP3 receptors localized in lipid rafts [56, 57]. Selective inhibition of gBK_Ca_ channels, for example, using compounds like paxilline or penitrem A, reduces glioma cell proliferation and migration without significantly affecting normal tissues [58, 59]. Immunotherapy strategies targeting gBK‐specific epitopes have also shown promise, enabling cytotoxic T lymphocytes to selectively destroy glioma cells [60, 61]. Additionally, combining gBK_Ca_ inhibitors with standard treatments like temozolomide (TMZ) may enhance antitumor efficacy, while reducing drug doses and side effects [61, 62]. This approach exploits the tumor‐specific expression of gBK_Ca_, allowing for more precise and safer therapeutic interventions.

### 3.3 BK_Ca_ channels in glioblastoma cell proliferation, migration, and invasion

**Fig. 1:**
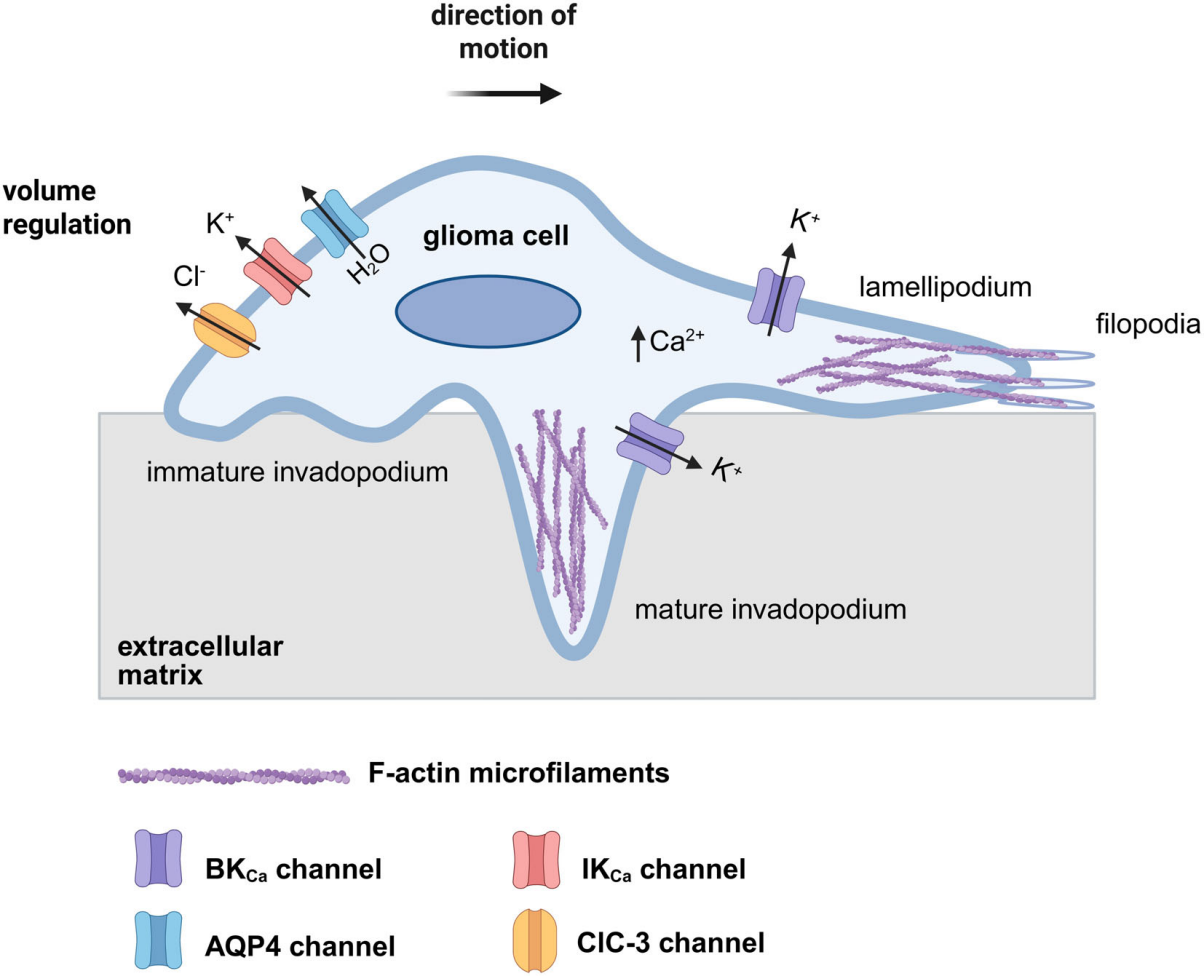
Schematic illustration of the ion transport and invasion of glioblastoma cells through extracellular matrix (ECM): Glioblastoma cells form actin‐rich structures, such as invadopodia, lamellipodia, and filopodia, to invade the neighboring ECM and move in a precise direction. These protrusions help degrade the ECM and promote cell motility. Additionally, glioblastoma cells possess an efficient volume regulatory mechanism, allowing them to migrate long distances through the brain. They can squeeze through narrow interstitial spaces by losing water and reducing cell volume. This is facilitated by the cotransport system, leading to Cl^−^ accumulation beyond its electrochemical equilibrium. Cl^−^, along with K^+^, serves as an osmolyte, and the efflux of Cl^−^, K^+^, and water through Cl^−^ channels (ClC3), K^+^ channels (IK_Ca_ or BK_Ca_), and aquaporin channels (AQP4) enables the cells to reduce their volume, promoting their invasive behavior. Prepared based on [53, 166, 167, 168] and created in BioRender.com.

The adhesion and motility of many cell types (including macrophages, dendritic cells, endothelial cells, vascular smooth muscle cells, osteoclasts, fibroblasts, myotubes) are partly regulated by the assembly of podosomes, specialized, actin‐rich structures of the cytoskeleton [63, 64]. They are highly dynamic, undergoing cycles of degradation and reassembly within seconds to minutes, and their formation is strictly calcium‐dependent [64, 65]. In invasive cancer cells, including glioblastoma, analogous structures are referred to as invadopodia, which support cancer spreading and are considered critical determinants of tumor invasiveness and metastatic potential [63]. By contrast, lamellipodia are flat, sheet‐like actin‐based protrusions at the leading edge of migrating cells; often, all these terms are incorrectly interpreted in the literature, which is worth emphasizing [66]. Oncochannels have been proposed to contribute to the dynamic changes in cell shape and volume required for invadopodia extension, as reviewed previously [53]. In glioblastoma cells, Cl^−^ channels have been localized at lamellipodia and shown to regulate migration in rat C6 and human glioma biopsy cells [67]. BK_Ca_ channels regulate the movement of ions and H_2_O, working alongside Cl^−^ channels, which is crucial for the regulation of cell capacity during migration and invasion. BK_Ca_ channels contribute to glioma migration, as demonstrated in human D54MG cells [9], and were detected at lamellipodia of U251MG glioblastoma cells [68]. A schematic illustration of the ion transport and invasion of glioblastoma cells through extracellular matrix is presented in Fig. 1. The interaction between BK_Ca_ channels and other ion channels, such as the sodium‐calcium exchanger (NCX), is critical for maintaining the ionic balance necessary for invadopodia formation [69]. The regulation of lamellipodia formation and glioblastoma cell migration depends on calcium homeostasis, in which both NCX and BK_Ca_ channels play critical roles. NCX contributes by shaping intracellular Ca^2+^ levels, and its inhibition reduces lamellipodia formation and migration in glioblastoma cells [70, 71]. Although direct studies on dual NCX–BK_Ca_ targeting are lacking, the involvement of calcium signaling in both pathways suggests potential synergy. In this context, BK_Ca_ channels may cooperate with NCX to fine‐tune ionic balance during migration, making their combined modulation an attractive therapeutic strategy in glioblastoma.

Plasma membrane BK_Ca_ channels facilitate the efflux of potassium ions, leading to cell membrane hyperpolarization and subsequent cell volume reduction. The extrusion of potassium ions, followed by an osmotically driven water efflux, leads to a decrease in cell volume (Fig. 1). This reduction is a prerequisite for invadopodia formation, allowing cancer cells to penetrate the extracellular matrix. It has been proposed that tumor cells may need to shrink to invade narrow spaces in the brain, so what is connected with shrinkage depends on the efflux of K^+^ through BK_Ca_ channels [52]. Additionally, cell migration and invasion in glioblastoma depend critically on Ca^2+^ signaling, acting through multiple mechanisms [11, 12]. While BK_Ca_ channels are one important effector of localized Ca^2+^ influx, additional pathways—such as calpain‐mediated ECM degradation, calmodulin‐dependent invadopodia formation, and TRPC1‐driven chemotaxis—also contribute to invasiveness [72, 73, 74]. These parallel calcium‐dependent mechanisms highlight that BK_Ca_ channels operate as part of a broader signaling network. Thus, calcium‐dependent potentiation of BK_Ca_ channels should be viewed within this complex landscape, where multiple Ca^2+^‐regulated pathways converge to promote glioblastoma progression.

In light of these findings, which highlight the involvement of BK_Ca_ channels in regulating cell growth and migration, it is unsurprising that different glioma cells (e.g. lines U87‐MG, U87‐MG Katushka, U251MG, D54MG, U373‐MG, 1321N1) exhibit overexpression of BK_Ca_ channels. This overexpression likely supports glioma's aggressive, invasive, and proliferative behavior, reinforcing that BK_Ca_ channels could play a critical role in tumor progression and may represent a valuable target for novel therapies (Table 1) [52]. It should be noted that the genetic and transcriptomic profiling of the U87MG cell line demonstrated that its DNA profile differs from that of the original donor tumor U87‐MG cell line. However, this cell line is still considered a *bona fide* human glioblastoma cell line and it continues to be widely used as a standard model in glioblastoma research [75]. Stegen *et al*. demonstrated that BK_Ca_ channel activity contributes to the invasive capabilities of glioblastoma cells by regulating calcium concentration, which affects cytoskeletal dynamics and cellular motility [76]. This is crucial for glioblastoma's ability to infiltrate surrounding brain tissue, a key factor in its malignancy. Additionally, BK_Ca_ channel blockers, such as paxilline, have been shown to significantly reduce cell migration, suggesting the therapeutic potential of targeting these channels. Furthermore, it was highlighted that BK_Ca_ channel inhibition decreases migration and impairs glioblastoma cells' resistance to chemotherapy, particularly under hypoxic conditions [77]. Hypoxia is a hallmark of glioblastoma, enhancing tumor aggressiveness. BK_Ca_ channels support cell migration and survival in these low‐oxygen environments, making them attractive targets for new therapeutic approaches. Hu *et al*. demonstrated that blocking BK_Ca_ channels increases reactive oxygen species levels in glioblastoma cells, reducing proliferation and enhancing sensitivity to chemotherapy [78]. Hypoxic conditions activate BK_Ca_ channels, which in turn promote cell survival and migration. It has therefore been proposed that BK_Ca_ channels contribute to the adaptation of glioblastoma cells to hypoxia by regulating intracellular Ca^2+^ levels [79, 80]. By targeting BK_Ca_ channels, it may be possible to disrupt hypoxia‐driven survival mechanisms, leading to more effective glioblastoma treatments. Such interventions offer a multifaceted therapeutic strategy: in addition to directly inhibiting cell proliferation and migration, BK_Ca_ channel blockers could enhance the efficacy of existing modalities, including chemotherapy and radiotherapy.

**Table 1:** Summary of the BK_Ca_ channels' role in glioblastoma across different cell lines and conditions.

Glioblastoma cell lines	Conditions	Role of the BK_Ca_ as oncochannel	Therapeutic implications	References
U251MG, U87MG	Ionizing radiation	BK_Ca_ channels promote migration and invasion, enhanced by radiation therapy	BK_Ca_ channel blockers may reduce invasion and improve radiotherapy outcomes	[76]
T98G, U87‐MG Katushka	Ionizing radiation	BK_Ca_ channels drive migration and brain infiltration postradiation	BK_Ca_ targeting could mitigate radiation‐induced migration	[82]
U87MG	Radiation therapy	Radiation activates BK_Ca_ channels, contributing to migration and resistance	Inhibition of BK_Ca_ channels may reduce radiation‐induced migration and resistance	[81]
U87MG	Exposed to stress from chemotherapy and radiation	BK_Ca_ channels enhance cell survival under chemotherapy and radiation stress	Blocking BK_Ca_ channels could sensitize cells to chemotherapy and radiation stress	[10]
U87MG	Hypoxia	BK_Ca_ channels regulate intracellular calcium to promote survival in hypoxic conditions	BK_Ca_ channel inhibition may disrupt hypoxic survival, leading to better treatment responses	[172]
U87MG	Hypoxia	Inhibition of BK_Ca_ channels reduces migration and chemoresistance under hypoxia	Targeting BK_Ca_ channels could reduce resistance to chemotherapy in hypoxic environments	[77]
U87MG	Lipid raft disruption	Reduction BK_Ca_ channel activity by disrupting functional association with IP3 receptors	inducing IP_3_R‐dependent calcium release, may provide additional insight into the function of BK_Ca_ channels in the context of glioma cell invasion	[56]
D54MG U251MG	A specific isoform of BK_Ca_ identified	A specific isoform of BK_Ca_ channels regulates cell volume and invasion	BK_Ca_ inhibitors may reduce growth by impairing cell volume regulation	[52]
U87MG, U251MG	Interaction with Kir_4.1_	BK_Ca_ channels and Kir_4.1_ facilitate invasiveness by modulating cell dynamics	Combining BK_Ca_ and Kir4.1 inhibition may improve treatment strategies	[34]
U251MG	Glioma cells with BK_Ca_ channel blockage	BK_Ca_ channel blockage increases ROS, reducing proliferation and enhancing chemotherapy sensitivity	BK_Ca_ channel blockers could increase chemotherapy effectiveness by inducing ROS	[78]
1321N1	Phloretin treatment	BK_Ca_ activation inhibits migration	BK_Ca_ activation could be a strategy to inhibit migration	[173]
U373‐MG	OP‐A treatment	Induction of paraptosis‐like cell death by decreasing BK_Ca_ channel activity	BK_Ca_ modulation by OP‐A could be exploited to induce glioblastoma cell death	[133]

### 3.4 Ionizing radiation, DNA damage response, and metabolic adaptation

Interestingly, ionizing radiation at a therapeutically relevant dose stimulates excessive migration of human glioblastoma cells *in vitro* and enhances brain infiltration, as was proved using an orthotopic glioma xenograft mouse model [81, 82]. Therefore, one of the most significant challenges in treating glioblastoma is its resistance to radiotherapy [83, 84]. It was demonstrated that ionizing radiation activates BK_Ca_ channels, promoting glioblastoma cell migration [81]. Ionizing radiation has been shown to increase the activity of BK_Ca_ channels through mechanisms involving calcium signaling. Specifically, ionizing radiation stimulates the activation of CaMKII, which in turn enhances BK_Ca_ channel activity, leading to increased cell migration in T98G and U87MG glioblastoma cells measured by transwell migration assay [81]. A recent review has summarized the emerging role of ion channels in the DNA damage response [50]. Beyond their contribution to enhanced motility, BK_Ca_ channels are also functionally linked to the DNA damage response. Importantly, recent work demonstrated that pharmacological inhibition of Ca^2+^‐activated K^+^ channels (BK_Ca_ and IK_Ca_) in patient‐derived glioblastoma stem cells enhances radiation‐induced DNA double‐strand break persistence and reduces clonogenic survival, thereby linking these channels to DNA damage repair capacity [49]. In line with this, the absence of the BK_Ca_ channel has been reported to weaken the cellular response to DNA damage, potentially making cells more susceptible to particulate matter (PM)‐induced genomic instability [51].

BK_Ca_ channels are known to be closely associated with voltage‐gated calcium channels (VGCCs). This spatial proximity allows for efficient coupling of calcium influx through VGCCs to the activation of BK_Ca_ channels [13, 85]. Studies have shown that BK_Ca_ channels can be activated by calcium influx from both extracellular sources and internal stores, such as the endoplasmic reticulum [85]. It was demonstrated that ionizing radiation can suppress BK_Ca_ channel activity in certain contexts. For example, in rat coronary endothelial cells, ionizing radiation was found to suppress outward potassium currents, which are mediated by BK_Ca_ channels, suggesting a complex regulatory mechanism that may involve both activation and suppression, depending on the cellular context [86, 87]. By targeting BK_Ca_ channels, it may be possible to limit the radioresistance of these tumors, improving treatment efficacy. It was demonstrated that U251MG glioma cell migration (the ability of cells to transverse a transwell barrier), is blocked by BK_Ca_ channel inhibition after a 5‐h incubation with BK_Ca_ blockers: 100 nM iberiotoxin and 2 μM paxilline [10]. This experiment models the spatial restrictions that cells encounter while migrating through confined spaces in the brain. At the molecular level, BK_Ca_ channels are activated by both voltage changes and increases in intracellular Ca^2+^ levels. However, in the absence of voltage changes, an increase in calcium alone cannot activate these channels, like small conductance channels (SK). This dual activation mechanism is particularly relevant in glioblastoma, where calcium signaling pathways are frequently dysregulated. BK_Ca_ and Kir_4.1_ channels have been implicated in regulating glioblastoma invasiveness, as highlighted by Brandalise *et al*. [34] and further supported by experimental findings in U251MG cells reported by Ratto *et al*. [68].

This combined activation facilitates cell volume and cytoskeletal organization changes, enabling tumor cells to migrate through the dense brain extracellular matrix. Given their involvement in key processes of glioblastoma progression, BK_Ca_ channels present a promising target for therapeutic intervention.

## 4 Mitochondrial BK_Ca_ channels in glioma cells

### 4.1 Discovery and distribution of mitoBK_Ca_ channels

**Fig. 2:**
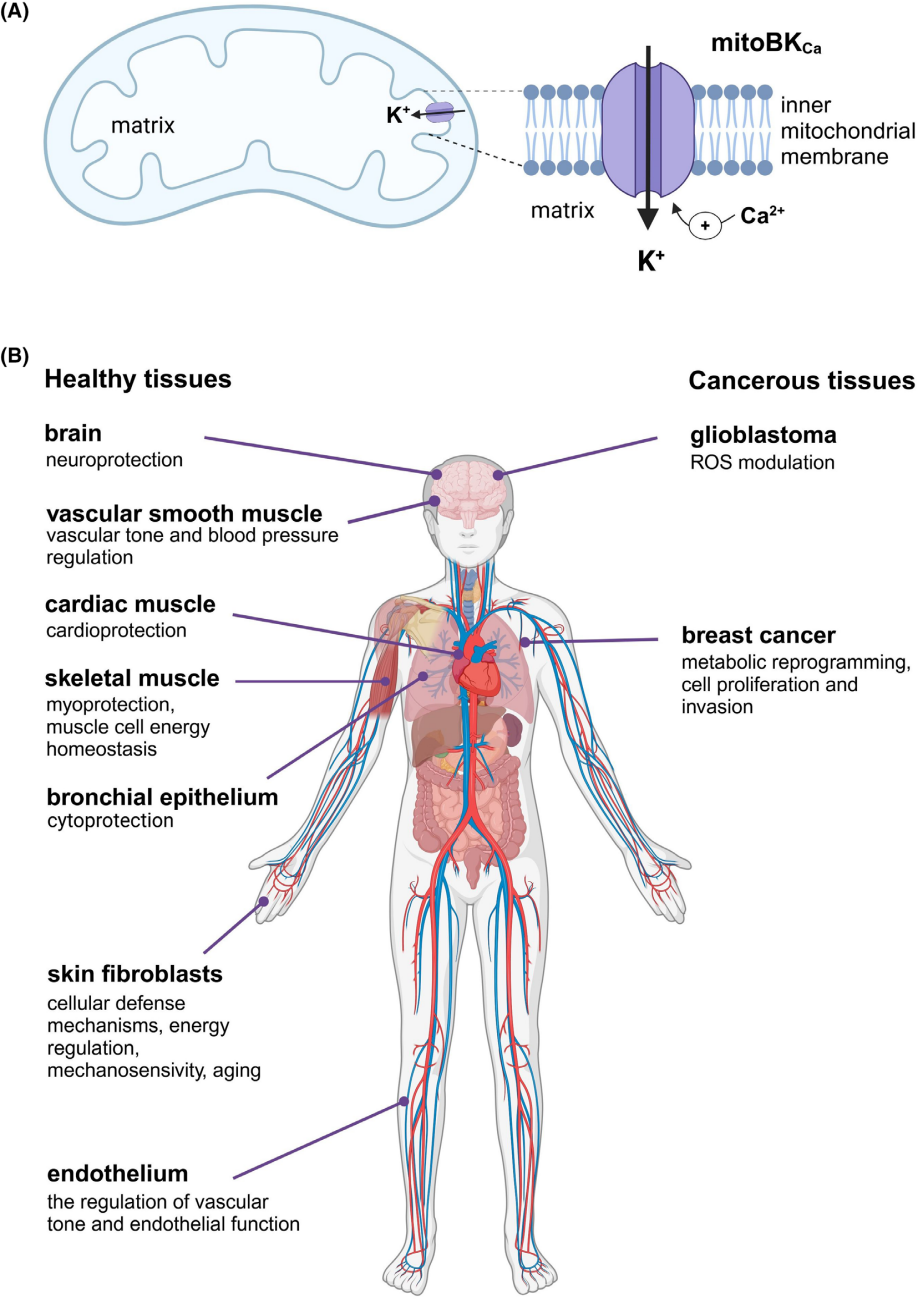
Distribution and function of mitoBK_Ca_ channels in various cell types. (A) The localization of the mitochondrial large conductance calcium‐activated potassium mitoBK_Ca_ channel, prepared based on [91]. (B) The roles of mitoBK_Ca_ in various cell types within healthy and cancerous tissues. In the brain, mitoBK_Ca_ channels contribute to neuroprotection [29, 92]. In vascular smooth muscle, these channels regulate vascular tone and blood pressure [94]. In cardiac muscle, mitoBK_Ca_ channels facilitate cardioprotection [93]. In skeletal muscle, they support myoprotection and maintain energy homeostasis [95]. MitoBK_Ca_ channels are involved in cytoprotection in bronchial epithelium, protecting respiratory cells from environmental threats [98, 99]. In skin fibroblasts, these channels play roles in cellular defense mechanisms, mechanosensitivity, aging, and energy regulation [100]. In endothelial cells, mitoBK_Ca_ channels are pivotal for regulating vascular tone and endothelial function [96, 97]. In cancerous tissues like glioblastoma, mitoBK_Ca_ channels modulate reactive oxygen species [101], while in breast cancer, they are involved in metabolic reprogramming, cell proliferation, and invasion [102]. Created in BioRender.com.

Importantly, BK_Ca_ channels are located not only in the plasma membrane but also in cellular organelles, including mitochondria (Fig. 2A). In 1999, Siemen *et al*. identified the mitochondrial isoform of BK_Ca_ channels known as the mitoBK_Ca_ channel in the human glioma cell line LN229, and later, this channel was found in U87 MG cells [88]. It has been shown that mitoBK_Ca_ channel properties were similar to the plasma membrane BK_Ca_ channel [89, 90]. The mitoBK_Ca_ channels are present not only in the mitochondria of glioma cells but also in many healthy and cancerous tissues [91] (Fig. 2B). The mitochondria of various healthy tissues, including the brain [29, 92], cardiac [93], smooth [94] and skeletal muscle [95], endothelium [96, 97], the bronchial epithelium [98, 99], and skin fibroblasts [100], contain the mitoBK_Ca_ channels. In cancerous tissues, they are present in glioblastoma [101] and breast cancer cells [102].

### 4.2 Functional roles of mitoBK_Ca_ in healthy and cancerous cells

Although plasma membrane BK_Ca_ channels are more abundantly expressed and regulate excitability and calcium signaling, mitochondrial BK_Ca_ channels—despite their lower expression—are essential for maintaining mitochondrial calcium balance, reactive oxygen species (ROS) control, and stress adaptation, reflecting their distinct but complementary contributions to glioblastoma cell physiology [68, 88, 92, 96].

mitoBK_Ca_ channels play crucial roles in both normal and cancerous cells, but their functions can differ significantly between these contexts. In healthy cells, mitoBK_Ca_ channels are involved in several key processes: activation of mitoBK_Ca_ channels modulates ROS production, which is crucial for protecting cells from oxidative stress [101, 103]. Also, it has been established that these channels maintain mitochondrial membrane potential and support efficient respiration, contributing to overall cellular energy homeostasis [103, 104].

### 4.3 Distinct behavior of mitoBK_Ca_ channels in glioma

In glioma cells, the function of mitoBK_Ca_ channels exhibits some distinct characteristics. Specifically, the activity of mitoBK_Ca_ channels in glioma cells is closely regulated by the mitochondrial respiratory chain. This regulation is crucial for maintaining mitochondrial function and ROS levels [101, 104]. Knockout studies in glioma cells lacking mitoBK_Ca_ channels have shown increased levels of mitochondrial ROS, indicating that these channels play a significant role in controlling oxidative stress in cancer cells [101]. Unlike in healthy brain cells, where mitoBK_Ca_ channels are inhibited under hypoxic conditions, in glioma cells these channels remain active and even increase their open probability during hypoxia. This adaptation may contribute to the hypoxia tolerance observed in tumor cells [79]. Glioblastoma cells frequently display mitochondrial dysfunctions, particularly in the context of *IDH1* mutations, which reprogram metabolism and impair mitochondrial respiration [105, 106, 107]. Reduced ATP production has been reported in *IDH1*‐mutant glioma cells, linking altered bioenergetics to impaired survival [108, 109].

### 4.4 Regulatory mechanisms of mitoBK_Ca_ activity

The mitoBK_Ca_ channel is activated after the entry of Ca^2+^ into the mitochondrial matrix, indicating that the channel's C‐terminal region, which contains the calcium‐sensing domain, is located within the mitochondrial matrix. These channels are regulated by various endogenous factors, including gasotransmitters like CO and H_2_S, heme, redox state, and phosphorylation by kinases [110]. Additionally, mitoBK_Ca_ channels have been reported to display mechanosensitive properties in glioblastoma cells [111]. However, mechanosensitivity is not unique to glioblastoma or to the mitochondrial isoform, as stretch‐activated BK_Ca_ channels have also been described in other cell types, such as ventricular myocytes [112] and cardiac tissue more broadly [113]. Thus, while glioblastoma mitochondria exhibit mechanosensitive BK_Ca_ activity, this phenomenon likely reflects a more general property of BK_Ca_ channels rather than a GBM‐specific feature. The above regulatory mechanisms indicate specific, mitochondrial pathways of mitoBK_Ca_ channels regulation. The complexity of mitoBK_Ca_ channel regulation suggests that it may be a crucial component in multiple signaling pathways that control mitochondrial activity in glioblastoma cells.

### 4.5 Structural topology and implications for glioblastoma

An important aspect that distinguishes mitoBK_Ca_ from its plasma membrane counterpart is its unique topological orientation within the inner mitochondrial membrane. While the general architecture of the channel is conserved (S0–S6 transmembrane helices and cytosolic RCK1/RCK2 gating domains), the spatial context is inverted: domains that would be extracellular in the plasma membrane instead face the intermembrane space, whereas the large Ca^2+^‐sensing C‐terminal tail resides in the mitochondrial matrix [79, 104]. This has profound functional implications in glioblastoma cells, where mitoBK_Ca_ channels integrate mitochondrial rather than cytosolic Ca^2+^ signals, thereby coupling matrix calcium dynamics to ROS regulation, oxidative phosphorylation, and apoptosis sensitivity. Such an arrangement may provide GBM cells with a survival advantage under stress conditions by allowing direct mitochondrial control over energy metabolism and redox balance. Thus, the inverted topology of mitoBK_Ca_ channels is not merely a structural peculiarity, but likely a critical factor contributing to glioblastoma progression.

### 4.6 Molecular identity and functional confirmation in glioma

Activation of the mitoBK_Ca_ channel induces potassium influx into the mitochondrial matrix, which causes depolarization of the inner mitochondrial membrane and an increase in the respiration rate. The K^+^ ion inflow into the mitochondrial matrix probably affects the influx of calcium ions. Ca^2+^ ions play a crucial role in mitochondrial metabolism, notably by regulating the activity of enzymes in the Krebs cycle [102]. Thus, the presence of the mitoBK_Ca_ channel appears to regulate mitochondrial metabolism. In cancer cells, this likely has significant implications for metabolic reprogramming. Pharmacological and biophysical properties suggested a strong molecular similarity between the mitoBK_Ca_ and the plasma membrane BK_Ca_ channels [114]. This led to the reasonable speculation that both channels were splice variants of the same gene product. Disruption of the *KCNMA1* gene in the U87 MG cell line resulted in loss of activity in mitoBK_Ca_ channels. This confirmed that one gene, *KCNMA1*, encodes plasma BK_Ca_ and mitoBK_Ca_ channels in U87 MG cells. Additionally, the presence of the mitoBK_Ca_ channel is crucial for adjusting ROS levels in glioblastoma cells, which strongly suggests its important regulatory role [101]. Specifically, the VEDEC splice variant has been shown to form functional channels in the inner mitochondrial membrane [90]. The biophysical properties of mitoBK_Ca_ channels, such as high conductance (~290 pS), voltage‐dependency, and Ca^2+^−sensitivity, are consistent with those of plasma membrane BK_Ca_ channels [90, 98]. These channels are modulated by various factors, including auxiliary β subunits and pharmacological agents [98, 115]. MitoBK_Ca_ channels play a crucial role in regulating mitochondrial functions such as membrane potential, respiration, and ROS generation [111]. Their activation is cytoprotective, particularly in cardiac cells during ischemia/reperfusion injury [90]. In glioblastoma cells, mitoBK_Ca_ channels exhibit unique gating dynamics influenced by splice variants and auxiliary subunits, which affect their voltage‐ and Ca^2+^‐sensitivity [116, 117]. The presence of these channels in glioblastoma cells suggests a role in cell survival and proliferation, potentially through mechanisms involving mitochondrial fission and fusion [111].

## 5 Pharmacological modulation of BK_Ca_ and mitoBK_Ca_ channels

Pharmacological modulation of BK_Ca_ and mitoBK_Ca_ channels involves various activators and inhibitors that influence their activity and physiological roles. The roles and mechanisms of these modulators are discussed in the following subsections according to their origin and functional properties. Although many of these compounds have been studied in cardiovascular, neuronal, or noncancer models, direct evidence of their relevance in glioblastoma is still limited. Nevertheless, these findings offer a conceptual framework for future research into potential therapeutic targeting of BK_Ca_ channels in GBM.

### 5.1 Natural activators of BK_Ca_ and mitoBK_Ca_ channels

**Fig. 3:**
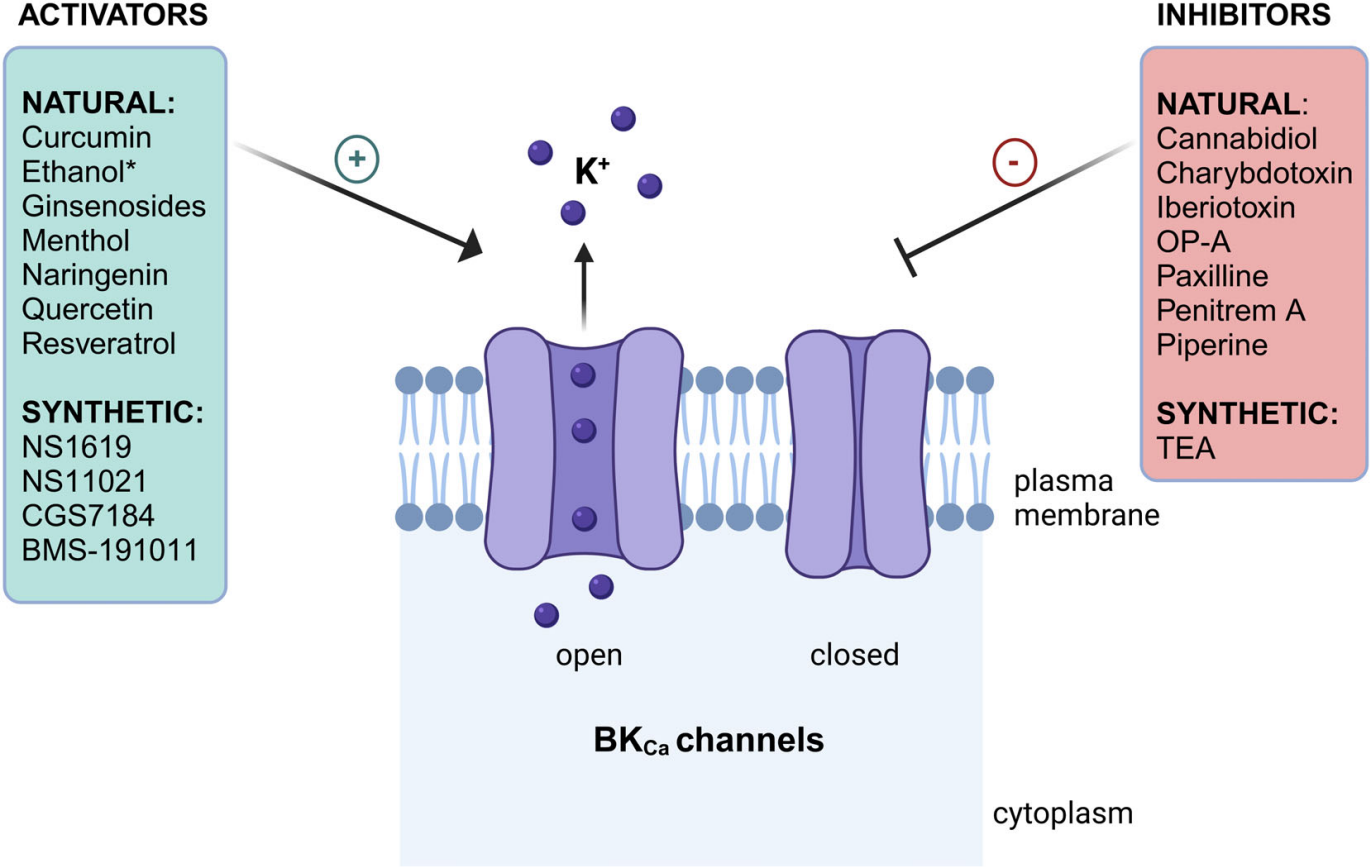
Exogenous natural and synthetic activators and inhibitors of BK_Ca_ channels. Natural activators: curcumin [125], ginsenosides [128], menthol [127], naringenin [119], quercetin [99], and resveratrol [126]. Synthetic activators: NS1619 [169], NS11021 [170], CGS7184 [139, 141], BMS‐191011 [140]. *Ethanol acts as an activator at low/moderate concentrations and an inhibitor at high concentrations (neurotoxic effects) [129, 130]. Natural inhibitors: cannabidiol [131], charybdotoxin [171], iberiotoxin, paxilline, penitrem A [171], OP‐A (ophiobolin A) [133], and piperine [132]. Synthetic inhibitor: TEA (Tetraethylammonium) [171]. Created in BioRender.com.

Due to the widespread presence of BK_Ca_ channels and their crucial role, extensive efforts have been made to identify specific activators among natural compounds and to develop synthetic alternatives. Flavonoids and other natural substances regulate BK_Ca_ activity alongside a group of synthetic modulators known as potassium channel openers [118] (Fig. 3). Naringenin and quercetin, which belong to the flavonoids, were described as plant‐derived activators of BK_Ca_ channels [99, 119, 120]. Naringenin (a flavanone obtained from citrus fruits) has been reported to activate BK_Ca_ channels in cardiac mitochondria, leading to cardioprotective effects in models of myocardial ischemia/reperfusion. This activation results in improved postischemic functional parameters and reduced myocardial injury, effects that are antagonized by the selective BK_Ca_‐blocker paxilline [120]. Naringenin also affects the conformational dynamics of mitoBK_Ca_ channels, influencing the stability and kinetics of channel conformations [121]. Quercetin (a plant flavonol) has been shown to increase BK_Ca_ currents in rat coronary artery myocytes, leading to hyperpolarization of cell membranes and increased frequency of spontaneous transient outward currents. This effect is mediated through the production of intracellular hydrogen peroxide [122]. Additionally, quercetin activates the mitoBK_Ca_ channel at much lower concentrations compared to its activation of the same channel in the plasma membrane [123]. This activation is significant, as it increases the channel's open probability, enhancing mitochondrial function and providing cytoprotective effects [124]. Other natural compounds have also been reported to modulate BK_Ca_ channel activity. Curcumin (curcuminoid of turmeric) and resveratrol (stilbenoid) act as activators, promoting cytoprotective responses [125, 126]. In heterologous systems, curcumin enhances BK_Ca_ channel activity and stabilizes channel expression in HEK293 and A7r5 smooth muscle cells via ERK1/2 signaling and proteasome inhibition [125]. Resveratrol has been shown to activate BK_Ca_ channels in rat cortical neurons, increasing their open probability and attenuating action potential firing [126]. Menthol in human glioblastoma T98G cells increases intracellular Ca^2+^ via TRPM8 activation [127]. The rise in Ca^2+^ levels indirectly stimulates BK_Ca_ channel activity, which in turn can influence cell migration [127]. Ginsenosides (gintonins) are additional natural activators [128], while ethanol exerts bidirectional effects, acting as both an activator and inhibitor, depending on concentration and subunit composition [129, 130]. Gintonin, a bioactive glycolipoprotein derived from ginseng, activates BK_Ca_ channels in the heterologous *Xenopus* oocyte systems in a concentration‐dependent manner [128]. Ethanol acts as a calcium adjuvant, modulating BK_Ca_ gating to influence neuronal excitability and vascular tone [129].

### 5.2 Natural inhibitors of BK_Ca_ and mitoBK_Ca_ channels

Conversely, several natural inhibitors have been identified. Cannabidiol (CBD) directly inhibits BK_Ca_ channel activity, as shown in HEK293 cells using patch‐clamp recordings. CBD reduces BK_Ca_ currents in a concentration‐dependent manner, through direct action that lowers unitary conductance and stabilizes the closed channel state [131]. Piperine, a plant alkaloid, has been shown to block BK_Ca_ channels in a subunit‐specific manner [132]. It inhibits specific BK_Ca_ subunit combinations (α and αβ₁/β₄), with selective inhibitory potency demonstrated in HEK293T cells [132]. The fungal metabolite ophiobolin A (OP‐A) reduces BK_Ca_ channel activity and induces paraptosis‐like cell death in human U373MG glioblastoma cells, as measured by the patch clamp technique [133]. Toxin peptides isolated from scorpion venoms, such as charybdotoxin and iberiotoxin [134, 135], along with paxilline, a fungal metabolite, have been reported as natural inhibitors of BK_Ca_ channels [123]. In human endothelial cells, the lack of a paxilline‐blocking effect on the mitoBK_Ca_ channel after its activation by quercetin was likely due to the spatial blocking of the paxilline binding site by quercetin [123]. The spectrum of these natural activators and inhibitors, together with synthetic modulators, is summarized in Fig. 3, which illustrates their opposing effects on BK_Ca_ channel activity.

### 5.3 Synthetic modulators: Openers and blockers of BK_Ca_ and mitoBK_Ca_ channels

Several synthetic compounds, such as NS11021 or NS1619, are known BK_Ca_ channel activators [136, 137, 138]. Additional synthetic openers include CGS7184 and BMS‐191011, which show strong activating properties but, like other potassium channel openers, suffer from limited selectivity and off‐target effects [139, 140, 141]. On the inhibitory side, tetraethylammonium (TEA) is a classical synthetic BK_Ca_ channel blocker that has been widely used in electrophysiological studies as a pharmacological tool [137]. Neuroprotective effects of BK_Ca_ channel modulators NS1619 and paxilline were observed in rat hippocampal neurons. Pharmacological activation of the BK_Ca_ channel decreased neuronal cell death induced by glutamate [142]. The potential mechanism of the neuroprotective action of potassium channel openers involves inhibiting ROS synthesis by the mitochondrial respiratory chain [114]. In glioma cells, NS004 and NS1619 were observed to affect mitochondrial function. These KCOs decreased the mitochondrial membrane potential and inhibited mitochondrial function due to the inhibition of complex I of the mitochondrial respiratory chain. However, the inhibition of mitochondrial functions by NS004 and NS1619 did not affect cell survival [143]. In human malignant glioma D54MG cells cultured under serum‐free conditions, pharmacological inhibition of glioma‐specific BK_Ca_ splice variants with iberiotoxin (100 nM IbTX) caused dose‐ and time‐dependent growth arrest by S‐phase cell cycle blockade and cell death, effects entirely negated in serum‐containing media [9]. However, the mechanisms behind this phenomenon remain unclear.

The pharmacological modulation of BK_Ca_ channels is fraught with challenges due to the specificity issues of current modulators and activators [144]. These challenges stem from off‐target effects, lack of structural data, variability in channel composition, and the impact of posttranslational modifications and alternative splicing. Addressing these issues requires a deeper understanding of BK_Ca_ channel structure and function, as well as the development of more selective and precise modulators in GBM models.

## 6 Current therapeutic strategies targeting BK_Ca_ channels in glioblastoma

Current therapeutic strategies against glioblastoma increasingly target BK_Ca_ channels to mitigate tumor growth, migration, and therapy resistance.

Recent evidence indicates that fungal metabolites, such as Ophiobolin A, can suppress BK_Ca_ channel activity, leading to cytoskeletal rearrangements and the induction of paraptosis‐like cell death in U373‐MG cells [133]. This inhibition leads to changes in the F‐actin cytoskeleton, reduced cell proliferation and migration, and induction of paraptosis‐like cell death, characterized by vacuolization due to mitochondrial and endoplasmic reticulum swelling and fusion [133]. These findings present a potential treatment approach to address GBM cells' innate resistance to proapoptotic signals.

Large hypoxic regions that are closely associated with tumor malignancy are a hallmark of GBM [145]. It has been demonstrated that low oxygen levels increase invasiveness and chemoresistance. A heavy hypoxic microenvironment upregulates BK_Ca_ channel activity, enhancing cell migration and chemoresistance to drugs like cisplatin [77]. Hypoxia in U87MG cells caused a functional increase in BK_Ca_ channel activity without affecting the expression of their plasma membrane. As demonstrated by transwell migration and wound healing assays, hypoxia enhanced U87‐MG cells' capacity to migrate; this effect could be countered by blocking BK channels. Hypoxia could cause chemoresistance to cisplatin in U87MG, according to toxicological tests. Inhibiting BK_Ca_ channels under hypoxic conditions can prevent these effects, suggesting that targeting BK_Ca_ channels could mitigate hypoxia‐induced aggressiveness in GBM [77].

BK_Ca_ channel targeting may be a helpful strategy to combat IR‐induced migration during radiotherapy, as radiation‐induced BK_Ca_ activation activates, and BK_Ca_ channel blockage inhibits this process. Ca^2+^ signals and BK_Ca_ channels are necessary for glioblastoma cell infiltration of the brain [82]. These signals program and drive the migration of glioblastoma cells, respectively. It has been demonstrated that ionizing radiation changes Ca^2+^ signaling, promotes glioblastoma cell migration, and induces the expression of the chemokine SDF‐1. Ionizing radiation increases BK_Ca_ channel activity, promoting GBM cell migration. Targeting BK_Ca_ channels with inhibitors like paxilline during radiotherapy can block IR‐induced migration and brain infiltration of GBM cells, suggesting a novel approach to enhance the effectiveness of radiotherapy [82]. Another approach involves examining whether potassium channel modulators can enhance the transport of anticancer drugs across the BBB. For example, the coinfusion of NS1619 with [^14^C]‐TMZ and Herceptin improved drug delivery to brain tumor cells [146]. Also, coinfusion with ATP‐sensitive potassium channel activator minoxidil sulfate resulted in improved and selective drug delivery to brain tumors [147].

Interestingly, another combination therapy was proposed based on auranofin (a thioredoxin reductase inhibitor) and CyPPA (SK channel opener) treatment [148]. By targeting thioredoxin reductases in conjunction with CyPPA to activate SK channels in neuro‐ and glioblastoma cells, cancer cell death was observed using the gold derivative auranofin. *In vitro*, neuroblastoma cells treated with auranofin and CyPPA experienced severe mitochondrial damage and increased auranofin‐induced toxicity. Specifically, respiration, mitochondrial integrity, and related energy production were compromised. Auranofin, combined with CyPPA, induced mitochondrial damage and enhanced toxicity in glioblastoma cells, suggesting a potential combinatory approach for the future to target BK_Ca_ channels.

Moreover, it has been determined that brain tumor‐initiating cells (BTICs) play a major role in the resistance mechanisms of GBM [149]. The necessity for developing new therapeutic approaches is highlighted by the fact that BTICs are elusive therapeutic targets that exist across the BBB. RNAi‐mediated targeting based on targeting multiple transcription factors that drive glioblastoma stem cell phenotypes using siRNA encapsulated in nanoparticles has shown potential in reducing tumor growth and extending survival in preclinical models. This strategy could also be adapted to target BK_Ca_ channels specifically.

Another approach focuses on the role of BK_Ca_ channels in cancer immunotherapy. Ge *et al*. described two potential T‐cell epitopes in the gBK_Ca_‐specific protein sequence [150]. T cells sensitized to gBK_Ca_ peptides can kill target glioma cells expressing gBK_Ca_ channels. On the other hand, it was observed that activation of the BK_Ca_ channel led to glioma cell swelling and vacuolization [151]. Disruption of internal potassium ion balance stimulated monocytes to paraptosis, and the mechanism included the mitochondrial pathway [152].

Combining K^+^ channel inhibitors with other treatments has shown potential. For instance, combining TMZ and TRAM‐34, a K_Ca3.1_ channel inhibitor, has reduced GBM cell migration, invasion, and colony formation while increasing apoptosis and survival in mouse models. This combination also affects the tumor microenvironment, reducing the toxic effects on neuronal cultures [62]. These findings point to a novel treatment strategy for malignant gliomas that targets glioma cell migration and proliferation. TRAM‐34 is a selective inhibitor of K_Ca3.1_ rather than a direct modulator of BK_Ca_. However, since K_Ca3.1_ and BK_Ca_ channels can functionally interact in shaping calcium signaling and membrane potential, pharmacological inhibition with TRAM‐34 may indirectly influence BK_Ca_‐dependent processes in glioblastoma cells [153].

These strategies highlight the potential of targeting BK_Ca_ channels in GBM to improve therapeutic outcomes by reducing tumor aggressiveness and overcoming resistance to conventional treatments.

## 7 Current challenges in developing BK_Ca_ channel‐targeted therapies for glioblastoma

The BBB restricts the delivery of many small and large molecules into the brain, making it difficult for chemotherapeutic agents to reach the tumor site effectively [154]. Active efflux transporters at the BBB further limit drug delivery by pumping therapeutic agents out of the brain, reducing their concentration at the tumor site. GBM's invasive nature means that tumor cells can reside in areas of the brain protected by an intact BBB, making it challenging to target these cells with systemic therapies.

GBM exhibits significant cellular and molecular diversity, which includes variations in gene expression, genetic mutations, and epigenetic modifications [155]. This heterogeneity is driven by clonal evolution, where different clones acquire distinct mutations over time, contributing to genetic variability. GSCs are believed to play a crucial role in maintaining this heterogeneity and driving tumor progression. The interaction between tumor cells and the surrounding microenvironment, including immune cells, stromal cells, and extracellular matrix components, further contributes to the heterogeneity [156]. This heterogeneity complicates the development of targeted therapies, as treatments effective against one subpopulation of tumor cells may not be effective against others [157, 158].

GBM cells often exhibit multidrug resistance mechanisms, rendering many therapeutic agents ineffective. This resistance is a significant hurdle in developing successful treatments, including those targeting ion channels like BK_Ca_ [157, 159, 160]. Pharmacological inhibitors of BK_Ca_ channels, such as paxilline and penitrem A, have shown antiproliferative effects on GBM cells. However, these effects were observed at concentrations higher than those needed to inhibit channel activity. This suggests that the inhibitors might act through off‐target mechanisms, complicating their use as specific therapeutic agents [58]. Moreover, GBM involves complex signaling networks that contribute to its aggressive behavior and resistance to treatment. Targeting a single pathway or ion channel may not be sufficient to produce a significant therapeutic effect due to the redundancy and adaptability of these networks [157, 160, 161]. Finally, developing relevant animal models that accurately recapitulate human GBM is crucial for preclinical testing of new therapies. Current models often fail to mimic human disease fully, limiting the predictive value of preclinical studies [158]. Ensuring that targeted therapies do not adversely affect normal brain tissue is critical. The potential for toxicity and adverse effects in healthy tissues remains a significant concern in developing BK_Ca_ channel‐targeted therapies [157, 162].

Disruption of mitochondrial ion homeostasis could represent a novel approach to overcoming cancer drug resistance. One proposed strategy uses a mitochondria‐targeting triphenylphosphine‐modified block polymer (MPTPP) to selectively deliver an artificial K^+^ channel molecule 5F8 to cancer cells' mitochondria [163]. This molecule forms a K^+^‐selective ion channel, disrupting ion homeostasis and causing mitochondrial dysfunction. This design may benefit drug‐resistant cancer therapy, preventing multidrug‐resistant cancer cells from apoptosis. Moreover, combined activation of artificial and natural origin ion channels for disrupting mitochondrial ion homeostasis toward effective postoperative tumor recurrence and metastasis suppression was proposed [164]. It was demonstrated that pharmacological manipulation of mitochondrial ion channels provides a promising approach to cancer therapy by disrupting intracellular ion homeostasis and influencing mitochondrial function and structure. To achieve this, an engineered mitochondrial‐targeted delivery system based on an amphiphilic mitochondrial‐targeting polymer (TMP) was utilized to codeliver an endogenous K_ATP_ channel agonist (dinitrogen oxide, DZX) and artificial K^+^ channel‐forming molecules (5F8) in animal models of breast cancer [163]. DZX and the synthetic compound 5F8 have been shown to increase K^+^ influx and trigger oxidative stress–induced apoptosis, especially when used in combination. These observations highlight that engineering pharmacological or synthetic modulators of ion channels can disrupt intracellular ion homeostasis to enhance tumor cell killing, suggesting that analogous strategies could in the future be explored for BK_Ca_ channels in glioblastoma therapy. Synthetic molecules capable of replicating the function of natural ion channel proteins hold tremendous potential as therapeutic agents; for instance, by promoting apoptosis or modulating autophagic pathways through alterations in intracellular pH or the induction of oxidative and osmotic stress [165]. Despite these promising prospects, our understanding of the direct correlation between ion transport and specific biological outcomes remains limited.

## 8 Conclusions and future directions

In conclusion, both BK_Ca_ and mitoBK_Ca_ channels are integral to glioblastoma pathophysiology, influencing tumor growth, survival, and resistance to treatment. Targeting these channels alone or combined with other therapeutic strategies could open new avenues for treating GBM, potentially improving patient clinical outcomes. While targeting BK_Ca_ channels presents a promising avenue for GBM therapy, overcoming challenges is essential for developing effective and safe treatments. Continued research and innovation are necessary to address these obstacles and improve therapeutic outcomes for GBM patients. Future research is needed to better understand the precise molecular mechanisms underlying BK_Ca_ and mitoBK_Ca_ in GBM and to develop effective and selective inhibitors that could translate into clinical benefits.

## Conflict of interest

The authors declare that they have no known competing financial interests or personal relationships that could have appeared to influence the work reported in this article.

## Author contributions

KMO: conceptualization, investigation, visualization, writing – original draft, writing – review and editing; KP: writing – original draft; SJ: visualization; BK: writing – original draft, writing – review and editing, funding acquisition; PB: funding acquisition, writing – original draft, writing – review and editing.
